# Growth and Puberty in Children with Inflammatory Bowel Diseases

**DOI:** 10.3390/biomedicines8110458

**Published:** 2020-10-29

**Authors:** Flavia Amaro, Francesco Chiarelli

**Affiliations:** Department of Pediatrics, University of Chieti, 66013 Chieti, Italy; dottflavia.amaro@gmail.com

**Keywords:** growth failure, delayed puberty, ulcerative colitis, Crohn’s disease

## Abstract

Inflammatory bowel diseases (IBD) are gastrointestinal tract pathologies of unknown etiology; they have an alternating trend, with active and silent phases. IBD are classified in two main forms: ulcerative colitis (UC) and Crohn’s disease (CD). Both have chronic and recurrent course, gastrointestinal symptoms, and extraintestinal manifestations. The altered immune response role seems to be important both in UC and CD. In the majority of cases, CD begins with abdominal pain, diarrhea, decrease in appetite, and weight loss; there can be also perianal fistulas, rhagades, and perianal recurrent abscesses. In addition, retarded growth and delayed puberty can precede the development of the disease or can even be predominant at onset. Growth retardation is found in 40% of IBD patients, but the underlying mechanism of this and other extra-intestinal manifestations are partially known: the main hypotheses are represented by malnutrition and inflammatory response during the active phase of the disease. The increased level of pro-inflammatory cytokines can influence growth, but also the onset of puberty and its progression. In addition, it could be essential to clarify the role and the possible effects of all the currently used treatments concerning growth failure and delayed puberty.

## 1. Introduction

Inflammatory bowel diseases (IBD) are relevant chronic diseases in children and adolescents characterized by inflammation across the entire gastrointestinal tract and by various clinical presentation and prognosis [1]. In the definition of IBD, three different subtypes are included: ulcerative colitis (UC), Crohn’s disease (CD), and IBD-unclassified [2]. CD affects the entire gastrointestinal tract from the mouth to anus, while UC affects the colon [3]. The incidence of pediatric IBD is also increasing in preschool children, and a rapid increase has been described in some geographic areas (about 7.2% per year) [4]. Pediatric IBD is familial in 19–41% of cases, in fact a positive family history is often present when CD is diagnosed before 11 years of age [3]. 

The pathogenesis of IBD still needs to be fully clarified, but the role of immune system dysfunction is well known [5,6]. In addition, some environmental factors and the modern human lifestyle might be involved in the increasing incidence of IBD [7].

A dysregulated immune response to intestinal bacterial antigens in an individual with a genetic predisposition is one of the explanations for the development of chronic inflammation inside the gut. The intestinal permeability increases because of the breach of the intestinal mucosal barrier, and consequently, there is great exposure of the immune system to antigens, mainly bacteria [8]. 

In addition, microbiota appears to be altered compared with healthy controls [9]. Microbiota is essential for pathogen protection, nutrition, metabolism, and the immune system, so it is possible that the dysbiosis could be related to IBD pathogenesis, even if a precise relationship has not been fully established yet [10]. In IBD patients, a decrease in bacteria with anti-inflammatory effects and an increase in bacteria that enhance the inflammation have been observed; in particular, it seems that there could be a lower level of Firmicutes and an increase in the presence of Proteobacteria and Bacteroides [11].

The first line of defense against the antigens is the innate immune system; in fact, at first, the wounds of intestinal mucosa are represented by the accumulation in the lamina propria of lymphocytes, plasma cells, Natural Killer (NK) cells, and macrophages by ulcers. Later, the macrophages create ulcers of the entire intestinal mucosa thickness [12,13].

**Figure 1:**
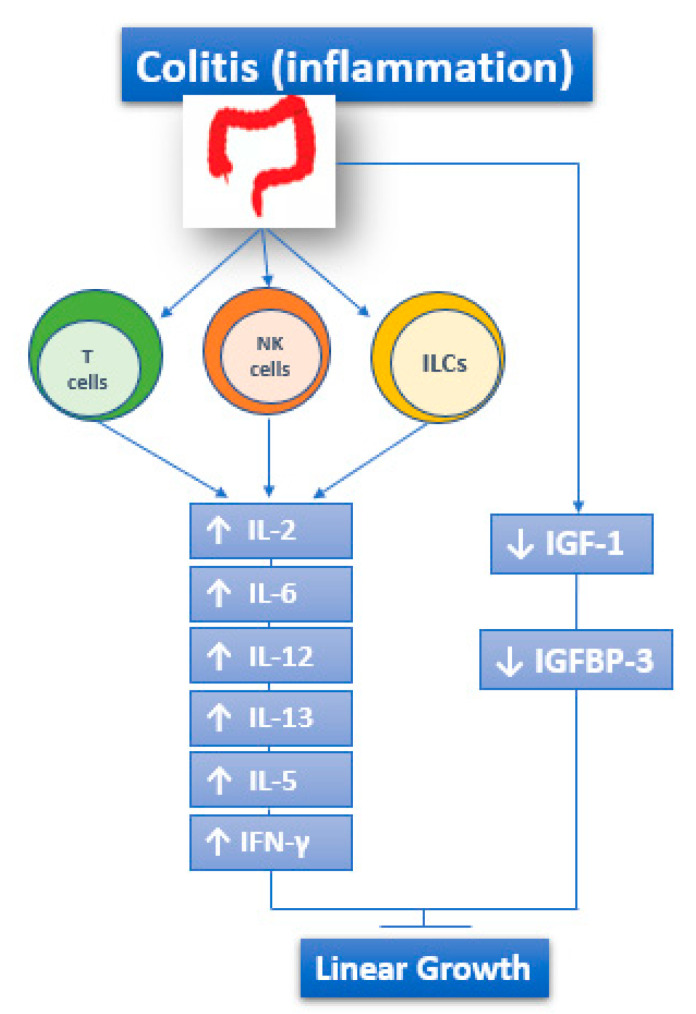
The role of immune response in IBD: both UC and CD are characterized by the increased and sometimes massive production of IL-2, IL-12, IL-6, IFN-γ, IL-5, and IL-13. ILCs: Innate Lymphoid cells.

The adaptive immune response is also involved in IBD and is responsible for the chronic inflammatory state. The activity of the adaptive immune system is represented by effector T lymphocytes, regulatory T lymphocytes, and innate lymphoid cells of intestinal mucosa [5,13,14]. Both UC and CD are characterized by the increased and sometimes massive production of Interleukin (IL)-2, IL-12, IL-6, Interferon γ (IFN-γ), IL-5, and IL-13 [15] (Figure 1). 

In addition, microRNAs (miRNA) seem to be involved in the immunopathogenesis of IBD because their dysregulation may result in excessive inflammation [16]. miRNAs are small, single-stranded, noncoding RNA molecules of 18–24 nucleotides that are involved in gene expression modulation at the posttranscriptional level and the current challenge is to identify a precise miRNA that controls a determined gene [17]. Research on IBD and miRNA started in 2008 when Wu et al. identified, for the first time, the miRNA profile in intestinal biopsies from IBD patients [18,19]. 

Since then, the research in this field has been stepped up with a focus on identifying their role as possible biomarkers in IBD diagnosis and as possible target therapy [20]. miRNAs have been shown to regulate specific genes associated with Crohn’s disease (CD) including nucleotide-binding oligomerization domain-containing protein 2 (NOD2), IL-6, and tumor necrosis factor (TNF), but further studies are needed to clarify their precise role and their use in IBD management [20].

Genomic studies have found about 200 loci that could be associated with genetic susceptibility to IBD and all of them encode proteins of the innate and adaptive immune system. In this field, the most important and frequent seems to be a polymorphism in the NOD2 gene [21].

All the cytokines and other mediators of inflammation have a role in the development of intestinal lesions, but they could also be involved in extra-intestinal manifestations of IBD such as cachexia, weight loss, growth failure, and pubertal delay [22].

IBD are systemic disorders and any organ and/or system could potentially be affected by them. Two types of extra-intestinal involvement could be identified: extra-intestinal manifestations (EIM) and extra-intestinal complications. The most frequent EIM are in the joints (peripheral and axial arthropaties), skin (erythema nodosus, pyoderma gangrenosum, Sweet’s syndrome, aphtous stomatitis), hepatobiliary tract (primary sclerosing cholangitis), and eye (episcleritis, uveitis); other EIM could affect rarely the lungs, the heart, the pancreas, and the vascular system [23]. Instead, the complications are caused by all immunogenic mechanisms that characterize the disease and so they are represented by malabsorption, micronutrient deficiencies, osteoporosis, kidney stones, peripheral neuropathies, and gallstones [23].

In general, the course of IBD, and in particular of CD, is more severe in females, but complications like growth failure and puberty delay seem to be more frequent in males [24].

The first-line necessary tests are represented by complete blood count, liver enzymes, albumin, C reactive protein, and/or erythrocyte sedimentation rate [25]. Other useful markers are the fecal calprotectin and lactoferrin, which in any case could be increased in several inflammatory conditions [26]. There are also serological markers like atypical perinuclear antineutrophil cytoplasmic antibody (pANCA) and anti-saccharomyces cerevisiae antibody (ASCA), which are particularly useful in determining the long-term prognosis [27]. Endoscopy is obviously fundamental: the ESPGHAN/NASPGHAN guidelines recommend total colonoscopy, upper endoscopy (esophagogastroduodenoscopy), multiple biopsies, and complete small bowel exploration [28]. Imaging technics such as computer tomography (CT), magnetic resonance enterography (MRE), and small intestine contrast ultrasonography (SICUS) are useful at diagnosis as well as to assess treatment efficacy and disease status [3].

The purpose of this review was to analyze the possible mechanisms that cause extraintestinal manifestations of IBD, particularly growth failure and delayed puberty, and to understand the effect of the IBD treatments on them.

## 2. Research Strategies

We compiled a review of the data described in the literature about ‘growth failure’ and ‘delayed puberty’ in IBD through the PubMed academic search engine. We analyzed data and articles found using the following keywords: “Inflammatory Bowel Disease in Children”, “Inflammatory Bowel Disease and Growth Failure”, “Growth Failure”, “Inflammatory Bowel Disease and Delayed Puberty”, “Delayed Puberty”, “Treatment of Inflammatory Bowel Disease”, “Inflammation process in Inflammatory Bowel Disease”, “Malnutrition in Inflammatory Bowel Disease”, and “Treatment of IBD and extra-intestinal manifestations”.

## 3. Growth Failure and Delayed Puberty in Inflammatory Bowel Diseases (IBD)

Approximately 25% of patients with IBD are diagnosed during childhood and adolescence, and the majority during puberty and pubertal growth spurt [29,30].

Symptoms and clinical features of IBD in children and adolescents, at onset and during the disease, are very similar to those in adult patients, but there are two extraintestinal manifestations that characterize IBD in children: growth failure and delayed puberty [31]. Based on studies in the 1980s, 1990s, and 2000s, average standard deviation scores of −0.94 to −1.30 for weight and −0.5 to −1.11 for height at CD onset are reported [32]. The bone development is affected by inflammation and is characterized by low trabecular and high cortical bone density and sometimes by decreased muscle mass. For these reasons, IBD children have high risk of osteopenia and osteoporosis in adulthood [33].

Despite many studies, the underlying mechanisms are not fully understood. The main hypotheses are represented by malnutrition and inflammatory response during the active phase of the disease. The increased level of pro-inflammatory cytokines can impair growth, puberty onset, and its progression [22,34].

All the possible risk factors for growth failure and delayed puberty in IBD are shown in Table 1.

### 3.1. Growth Failure

Growth failure can be defined as a static height below the third percentile or a z-score below −2 standard deviations (SD). Another parameter that can be used to evaluate growth problems is height velocity, expressed as a percentile or a SD score according to gender and age [35,36].

Growth is a marker of health in children and adolescents and growth failure is considered as one of the most important complications of IBD in children [37]: it has been reported in 15–40% of patients with onset of CD in childhood and in 3–10% of patients with onset of UC in childhood, more commonly in males [38,39].

In the Sex Differences in Statural Growth Impairment in Pediatric Crohn’s Disease study (also known as the Growth Study), a poorer height growth in males was described: the data suggest that height gain is lower in males than females with skeletal maturation [40]. 

Children with IBD usually report abdominal pain; they may have malabsorption due to mucosal damage and increased energy requirement because of the inflammatory state. The result is a lower caloric intake and consequently a malnutrition state (with micronutrient deficits—iron, vitamin B12, folate, vitamin D, zinc, other fat-soluble vitamins) that might further impair growth and puberty [41].

Concerning iron deficiency, it is a frequent nutrient deficiency found in adult and pediatric IBD patients and it represents a consequence of malabsorption or intestinal bleeding or dietary restrictions [42]. This type of deficiency can lead to a status of anemia, which is influenced by cytokines (like TNF-α) and by hepcidin. In these situations, hepcidin increases and induces the ferroportin degradation (ferroportin is an enterocyte iron transport protein): in this way, inflammation cytokines may substantially interfere with iron absorption [42]. Vitamin D is very important for normal absorption of calcium and normal bone mineralization [42]. There are insufficient studies on the precise role of vitamin D in IBD in children, but it is probable that it could influence the severity and the course of IBD [43]. Normal bone metabolism could also be affected by vitamin K deficiency, which in some studies on adult patients has been reported with modifications of bone resorption [42,44]. Another important micronutrient is zinc: this is absorbed in the small intestine and its deficiency could cause some symptoms including diarrhea and growth failure. Low serum zinc levels have been reported in adolescents with CD compared to controls [42,45].

In addition, Ballinger et al. [46] reported anorexia and poor appetite in rats with experimental colitis as a consequence of an increased release of serotonin from the hypothalamus. Furthermore, IL-1 may influence hypothalamic activity and delayed gastric emptying, especially in CD patients [47,48,49].

**Figure 2:**
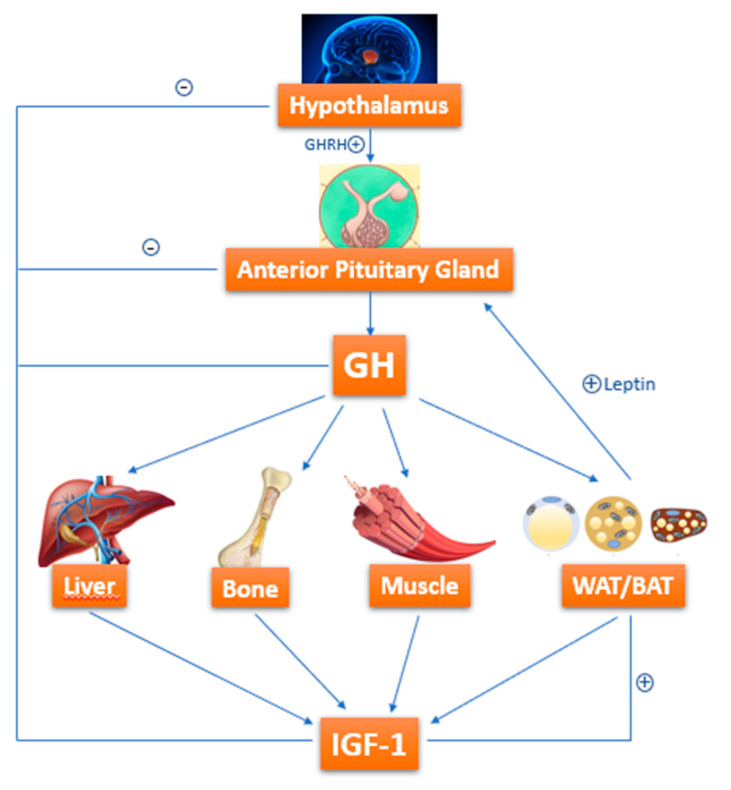
GH-IGF-1 axis: how growth hormone (GH) acts on target organs/tissues, leading to Insulin like Growth Factor-1 (IGF-1) production.

Growth hormone (GH)-resistance is also held liable for growth failure in IBD [50,51]. GH is a 191-amino-acid-long polypeptide secreted by somatotropes, which are cells of the anterior pituitary gland [52]. GH is secreted under control of growth hormone releasing hormone (GHRH) and somatostatin, which interact with each other and with several other factors to determine the pulsatile pattern of GH release. In normal conditions, GH stimulates Insulin like Growth Factor-1 (IGF-1) production by the liver. IGF-1 is fundamental in skeletal growth during puberty and bone health during one’s entire life; it increases tissue formation by acting directly and indirectly on target cells and is a crucial mediator of bone growth [52] (Figure 2).

IGF-1 enhances the growth of long bones through the stimulation of proliferation and hypertrophy of chondrocytes of the growth plates [53]. Growth plates in colitis-induced rats are characterized by the inhibition of proliferation and differentiation of cells responsible for growth: these rats seem to have a smaller proliferative zone in their growth plates and reduced terminal hypertrophic chondrocytes zone [54].

Circulating IGF-1 levels are decreased in active CD patients: they depend on several interlacing factors as nutritional status, disease activity, cytokines circulating levels, including tumor necrosis factor (TNF) and IL-1 [55]. Among these factors, inflammation seems to have a predominant role: in a recent study, Ballinger et al. analyzed the contribution of undernutrition and inflammation to growth deficit, comparing rats with colitis, healthy free-feeding controls, and a group of animals pair-fed (healthy animals whose daily food intake is matched to the colitis group) to those with colitis. Growth deficit was evident in pair-fed rats compared with healthy free-feeding controls, confirming the important negative effect on growth; in this study, by including a pair-fed group, the authors have tried to separate the effects of undernutrition (occurring in colitic and pair-fed groups in the same way) from inflammation (occurring only in the colitis group) on linear growth. The linear growth was further reduced in the colitis group, suggesting that inflammation may affect by itself growth (or in any case it can enhance the effects of undernutrition). In addition, in these rats, there was a negative correlation between plasma GH and IGF-1 levels, suggesting that there could be an inflammation-induced hepatocyte resistance to GH stimulation [29,56]. Gupta et al. [57] showed that IGF-1 levels were lower in males compared to females and that sex differences in growth failure did not depend on diagnosis timing and on timing of pubertal growth spurt.

In addition, children with CD present normal GH secretion with decreased circulating IGF-1 levels, and GH levels were also normal when they have been measured in urine or after stimulation tests: this evidence confirms a possible GH resistance [58,59,60].

The role of cytokines can be expressed in four different ways: (1) They might interfere with signal transduction of GH in the liver; (2) there can be a variation of IGF binding protein concentrations; (3) they can have direct effects on growth plate; and (4) cytokines may also interfere with the signal transduction of GH in growth plate chondrocytes [50]. 

Another hypothesis that is connected with altered IGF-1 function concerns the IGF binding proteins (IGFBPs): indeed, IGF-1 binds to seven different IGFBP and its bioavailability depends on them. The proteins themselves are influenced by the inflammatory status. Among them, the main one is IGFBP-3, and its circulating levels are decreased during active phase of CD and return to normal during the remission phase [61,62,63,64] (Figure 1).

Several studies have focused on susceptibility genes for IBD in general and for growth failure specifically [50]. The mainly correlated CD genes seem to be NOD2/CARD15, but they do not influence growth anyway [65].

Gene correlation has been described by several authors: Lee et al. [66] reported a significant association between growth failure and a polymorphism in the dymeclin gene DYM, after an analysis of 951 subjects with IBD; Russel et al. [67] showed a possible association of growth impairment with OCTN 1/2 variants within the IBD5 locus. Further studies are needed to clarify this important topic. 

Another important observation is that the lapse of time between the onset of symptoms and the diagnosis correlates with the severity of growth failure [68,69]. Poor growth (but also BMI and weight), measured by Z scores, persists at follow-up in the very early onset-IBD patients compared with older children who presented growth improvement during follow-up.

Furthermore, according to another hypothesis, growth retardation could be correlated with the site of the inflammation: patients with jejunal disease have been observed to have more severe growth impairment because they refer less specific symptoms than those with colitis and so the diagnosis might be substantially delayed [32,68].

Finally, IGF-1 production is also affected by glucocorticoid therapy [70,71,72].

Several studies have investigated the final adult height in patients with childhood-onset IBD: most of them have limitations such as a small number of patients, highly selected study populations, too short study periods, lack of consideration of parental height. The latter limitation is particularly significant because it is well known that parental height is one of the most important determining factors of adult height. A considerable number of IBD subjects will be shorter [73,74]; instead, other data show that the attained adult height is reduced only in a small number of patients with CD, but not in those with UC [75,76].

In any case, Mouratidou et al. [2] reported that most patients with childhood onset IBD had a low attained adult height, but the final height only seemed to be modestly lower than that of healthy peers and siblings. Therefore, high levels of severe inflammation markers are associated with reduced final adult height, so, perhaps, in these cases, patients have to be treated earlier or in a more aggressive way.

### 3.2. Delayed Puberty

Delayed puberty is frequently found in adolescents with inflammatory chronic disease [77] and it is particularly common in females with CD. It is defined as the absence of breast development in girls or testicular enlargement in boys that is 2.5 standard deviations below the population mean [78].

The underlying mechanisms are not fully understood: even in this case, pro-inflammatory cytokines such as IL-1 and TNF-α could play a relevant role [79]. Indeed, they inhibit the production of sex steroids through a direct action on gonads or through the suppression of gonadotropin-releasing hormone (GnRH) secretion [80]. In fact, inflammation can have an adverse effect on sex hormone levels and inflammatory markers seem to correlate with testosterone levels in males, but there is no correlation with estradiol levels in females [57]. 

Furthermore, some studies have assumed that leptin could have a role: it can represent a puberty trigger and its levels can be decreased in IBD due to malnutrition status and fat mass loss [81,82]. In any case, other studies have disapproved these theories: leptin does not seem to be a mediator of puberty delay [83,84]. Another point worthy of attention is that pubertal delay may potentially decrease bone mineralization and affect quality of life in children who realize that their sexual maturation is different from their peers [85].

## 4. Treatment of IBD and Its Effects on Growth Failure and Delayed Puberty

The goals of the treatment of IBD in children have changed over the last few years. In the past, the only purpose was to reduce symptoms, since other options were limited. Nowadays, because of new therapies and technologies, the natural history of the disease can be definitely modified and substantially improved. Therefore, the current goals of treatment are to eliminate symptoms and improve quality of life, recover normal growth, and reduce complications [86].

The major aim of all IBD treatments is inflammation control during the acute phase, but also during the remission phase, but another important aspect is the nutritional assessment that should include dietary history and symptoms check; in addition, it is also essential to do regular nutritional laboratory tests (25OHD, calcium, serum iron, ferritin, transferrin total iron-binding capacity (TIBC) and transferrin percent iron saturation, zinc, folate, vitamin B12, fat-soluble vitamins) [42]. 

There are two ways to improve growth: a direct way through hormones and growth factors and indirect through the improvement of inflammation.

In children, IBD arise in pre-pubertal period or in the few years before, so it is straightforward that beginning a proper treatment as soon as possible is fundamental in order to reach a good outcome in adult age, especially concerning height [50].

All treatments of IBD and its effects on growth failure and delayed puberty are shown in Table 2.

### 4.1. Glucocorticoid Therapy

Glucocorticoid therapy is very effective in reducing inflammation and is widely used in moderate and severe acute flares in IBD patients, even if its broad spectrum of adverse effects concerning growth and bone metabolism is well known [87]. Even small doses of corticosteroids (3-5 mg/m^2^) can impair the linear growth in pre-pubertal children [88]; budesonide seems to allow a better preservation of the bone mineral density compared with prednisolone [89]. In any case, budesonide is less effective than prednisolone and should be used only in mild to moderate pediatric CD, particularly when the terminal ileal and right colon are involved [90].

Corticosteroids may affect linear growth through several mechanisms: they may cause (together with the disease) suppression of osteoblastogenesis, favoring osteoblast and osteocyte apoptosis, and reducing bone formation; they inhibit calcium absorption through the gut mucosa and favor calcium excretion in urine [91,92,93,94]. Furthermore, glucocorticoids interfere with the GH/IGF-1 axis, reducing the expression of GH liver receptors and decreasing IGF-1 production, but they also have this similar effect on the growth plate [70,95].

In any case, it is unclear if children treated with corticosteroids during puberty achieve a lower final adult height (compared with children not treated in this way) because of the therapy itself or because of the inflammation persistence [96].

### 4.2. Aminosalicylates

Aminosalicylates have a topical anti-inflammatory effect on the intestinal mucosa through binding to the nuclear peroxisome proliferator-activated receptor-γ, which inhibits cytokines and the production of inflammation mediators [29]. There are oral formulations that release the active moiety 5-aminosalicylic acid (5-ASA) in the ileum and colon or topic formulations [97]. 

Currently, there are insufficient studies on the use of aminosalicylates in children, particularly with regard to their possible effects on growth [29]. 

### 4.3. Immunosoppressive Drugs

Immunosuppressive drugs such as azathioprine and 6-mercaptopurine interfere with the normal metabolism of purine. A multicenter, randomized clinical trial demonstrated that therapy with these drugs, in particular, 6-mercaptopurine, within the first eight weeks of the diagnosis reduces corticosteroid exposure and improves the maintenance of clinical remission in children with CD [86,98].

Immunosuppressive therapy seems to ensure clinical and biological remission in 50% of patients with CD through mucosal healing [99,100]. Therefore, these drugs could be useful to contrast growth failure because of their anti-inflammatory effect, but also because they reduce the need for glucocorticoids [101,102]. 

### 4.4. Methotrexate (MTX)

Methotrexate (MTX) has immunosuppressive and anti-inflammatory effects because it is able to inhibit the pro-inflammatory cytokines and also enhance the anti-inflammatory cytokine (like IL-10) and adenosine. In addition, MTX interferes with the expression of adhesion molecules, and, in turn, reduces lymphocyte recruitment in the intestinal mucosa [103]. MTX represents an alternative treatment for patients who are unresponsive or intolerant to azathioprine/6-MP. Even if some studies have already analyzed the MTX effects on growth, it is well known that it allows for a reduction in glucocorticoid use (as the other immunosuppressive drugs), but additional data are necessary to further clarify the beneficial effects on extra-intestinal IBD manifestations [29].

### 4.5. Exclusive Enteral Nutrition (EEN)

Exclusive enteral nutrition (EEN) is defined as the supply of 100% of caloric needs by liquid formula and its effectiveness on clinical remission is comparable with corticosteroid therapy in children with CD [104]. EEN contributes to adjust micronutrient and caloric deficiencies and it is usually administered for 6–8 weeks with polymeric formulas that are more palatable and effective [105].

A positive effect on growth failure from EEN could derive from an increase of IGF-1 and IGFBP-3, thanks to a reduction of the inflammatory state and an improvement of nutritional status [64]. In addition, EEN might allow a “bowel rest”, decreasing gut activity and the production of pro-inflammatory mediators, modifying intestinal flora, restoring nutritional deficits and regaining caloric intake [29]. Pigneur et al. conducted a randomized prospective trial in children with CD: both steroid and EEN induced clinical remission, but patients with EEN-induced remission had a higher rate of mucosal healing and a different gut microbiota [63,64,106].

In any case, EEN could not be a long-term maintenance treatment and so there are other dietary intervention forms that have to be considered: partial enteral nutrition (PEN) or cyclic EEN. Wilschanski et al. reported that linear growth improves after providing nocturnal supplementary enteral nutrition. A Canadian prospective study reported height and weight gain in a group of patients treated with intermittent elemental EEN compared with the controls [107,108]. In fact, recent ECCO/ESPGHAN consensus guidelines describe the intermittent use of EEN or PEN as potentially positive for improvement in linear growth in children with CD [109].

### 4.6. Biologic Drugs

Some studies have investigated the use of biologics in children with IBD and their effect on growth failure. The most used biologic drug is infliximab, a chimeric monoclonal antibody targeting TNF-α [110].

In childhood and adolescence, 88% of CD patients respond to infliximab with 56% in remission at one year; 73% of UC patients respond to it with 39% in remission at one year [111,112]. Adalimumab has also demonstrated efficacy in moderately to severely active CD in children [113]. In three retrospective studies, there was weight gain after infliximab therapy, but no significant changes in linear growth were reported [114,115,116]. Instead, other studies have shown a recovery of normal linear growth in children treated with biologics [117,118,119,120]. The relationship between treatment with infliximab and growth improvement involves several factors such as clinical response to treatment, stage of puberty, and degree of growth failure. In any case, biologic drugs induce mucosal healing, inhibit the inflammatory process, and reduce glucocorticoid use [121,122]. In addition, children who had never consumed glucocorticoids seemed to have greater beneficial effects on growth by biologics administration [118]. Further studies are needed to understand the effects of other biologics such as adalimumab or the recent certolizumab, which has not been approved by EMA. 

### 4.7. Growth Hormone

Growth hormone is currently used in children with growth problems such as growth hormone deficiency, constitutional short stature, and Turner syndrome. It has no direct effects on the pathological mechanism of IBD, so it is certainly not a first-line treatment [50]. 

The available data show evidence of the efficacy of recombinant human GH (rhGH) treatment concerning height velocity over a short- to medium-term follow-up, but GH therapy does not improve disease activity and pro-inflammatory cytokines; long-term follow-up data are definitely lacking [123,124]. 

The effects of GH in the treatment of active CD seem to depend on the improvement of the metabolic status of the patients since it enhances protein synthesis, intestinal absorption, and the formation of lean body mass [125].

Additional studies are needed to further evaluate the possible utility of GH therapy in IBD, especially its role in growth failure.

### 4.8. Surgery Options

The surgery option could also have positive effects on growth, especially in prepubertal children, in fact, growth resumption, depending on surgery, is extremely insufficient once puberty has started. The surgical options are intestinal resection or strictureplasty or both. In general, surgery is a “last resort” option that is implemented in the case of the failure of other treatments [126]. Strictureplasty can be an effective technique in preventing small bowel, weight gain, and improving gastrointestinal symptoms [127]. Furthermore, in children with delayed puberty, intestinal resection may lead to normal puberty and normal growth spurt within one year of surgery [128]. In any event, further studies are needed to better understand the possible benefit of surgery on growth failure in IBD children, both in the pre-pubertal and pubertal stages.

Finally, the resolution of inflammation and malnutrition should lead to a “catch-up” growth phase, during which the growth rate is accelerated and the child could reach the target height. In any case, in a severe malnutrition condition, the “catch-up” phase is weak and so the target adult height is not reached [129].

## 5. Conclusions

Measurements of height, weight, and pubertal stage are fundamental in children and adolescents; in both children with a certain IBD diagnosis and in children with a suspicion of these types of diseases, measurements are of paramount importance. These diseases affect the quality of life and growth failure and delayed puberty represents important complications that should be detected promptly in order to improve the outcome in adulthood.

The underlying mechanisms of these complications are not fully clarified, but there are several evidences that reveal a predominant role of malnutrition status and of the inflammatory process, especially for Crohn’s disease. Therefore, proposal of IBD therapy should aim for mucosal healing and reducing gastrointestinal symptoms, but also to contrast malnutrition and inflammation. 

It could be useful to find out possible serological markers (for example, among the multiple involved miRNA) that could help to make an early diagnosis and therapy management. Another interesting point is the one with regard to genomics: it could be desirable to detect specific predisposing genes that can allow for early intervention in the course of the disease and the evolution of complications. 

The frequency of growth failure and delayed puberty in IBD does not seem to have changed over the last 25 years despite new available treatments, so it will be essential to identify some new targeted medical treatment to solve this particular problem, considering other points as possible sex differences or patient’s age at onset of IBD.

In summary, all the treatment options seem to be useful in improving growth and pubertal development through different mechanisms, with the exception of glucocorticoid drugs. Further studies are therefore needed to clarify the pathophysiological aspects of growth failure and delayed puberty and all possible positive effects of the different therapies, but also to develop sex-specific prediction models in order to find patients at the highest risk for growth impairment and to address them toward appropriate early therapies. All these considerations will be very important, also considering how much these types of complications could affect the short-term and long-term quality of life and outcomes of children and adolescents with IBD.

## Figures and Tables

**Table 1:** Possible risk factors for growth failure and delayed puberty in inflammatory bowel diseases (IBD).

Probable Risk Factor	Correlated Factors/Mechanisms of Action
Malnutrition state	Malabsorption Lower caloric intake Anorexia and poor appetite Delayed gastric emptying
GH-resistance	Circulating IGF-1 decreased levels Inflammation-induced hepatocyte resistance
Cytokines increased levels	Interference with GH signal transduction in the liver Interference with GH signal transduction in growth plate chondrocytes Variation of IGF binding protein concentrations Direct effects on the growth plate
Susceptibility genes	Dymeclin gene DYM Gene OCTN (1/2 variants within IBD5 locus)
Inflammation site (Jejunal disease)	Type of disease with less specific symptoms than in colitis one and so the diagnosis might be delayed
Inhibition of the sex steroids production by cytokines	Direct action on gonads Suppression of GnRH secretion

**Table 2:** Treatment of IBD and its effects on growth failure and delayed puberty.

Therapy	Effects
Glucocorticoid therapy	Adverse effects on growth and bone metabolism ➢ Suppression of osteoblastogenesis ➢ Inhibition of calcium absorption ➢ Interference with GH/IGF-1 axis
Aminosalicylates	Further studies are needed to clarify their role
Immunosuppressive drugs	➢ Clinical and biological remission ➢ Reduction of glucocorticoids use
Exclusive enteral nutrition (EEN)	➢ Clinical remission ➢ Decrease of gut activity and of pro-inflammatory mediators production ➢ Increase of IGF-1 and IGFBP-3
Biologics (infliximab-Adalimumab)	➢ Weight gain ➢ Mucosal healing ➢ Inhibition of the inflammatory process ➢ Reduction of glucocorticoids use
Growth hormone	➢ Enhancement of protein synthesis ➢ Improvement of intestinal absorption ➢ Enhancement of lean body mass formation (Further studies are needed)
Surgery option (Strictureplasty-Intestinal resection)	➢ Small bowel prevention ➢ Weight gain ➢ Improvement of gastrointestinal symptoms (Further studies are needed)
